# Evaluation of adjective and adverb types for effective Twitter sentiment classification

**DOI:** 10.1371/journal.pone.0302423

**Published:** 2024-05-01

**Authors:** Syed Fahad Ali, Nayyer Masood

**Affiliations:** Capital University of Science & Technology, Islamabad, Pakistan; Kitami Institute of Technology, JAPAN

## Abstract

Twitter, the largest microblogging platform, has reported more than 330 million active users in recent years. Many users express their sentiments about politics, sports, products, personalities, etc. Sentiment analysis has emerged as a specialized branch of machine learning in which tweets are binary-classified to provide sentimental insights. A major step in sentiment classification is feature selection, which primarily revolves around parts of speech (POS). Few techniques merely focused on single features such as adjectives, adverbs, and verbs, while other techniques examined types of these features, such as comparative adjectives, superlative adjectives, or general adverbs. Furthermore, POS as linguistic entities have also been studied and extensively classified by researchers, such as CLAWS-C7. For sentiment analysis, none of the studies conceptualized all possible POS features under similar conditions to draw firm conclusion. This research is centered on the following objectives: 1) examining the impact of various types of adjectives and adverbs that have not been previously explored for sentiment classification; 2) analyzing potential combinations of adjectives and adverbs types 3) conducting a comparison with a benchmark dataset for better classification accuracy. To assess the concept, a renowned human annotated dataset of tweets is investigated. Results showed that classification accuracy for adjectives is improved up to 83% based on the *general superlative adjective* whereas for adverbs, *comparative general adverb* also depicted significant accuracy improvement. Their combination with *general adjectives* and *general adverbs* also played a substantial role. The unexplored potential of adjectives and adverb types proved better in accuracy against state-of-the-art probabilistic model. In comparison to lexicon-based model, proposed research model overruled the dependency of lexicon-based dictionary where each term first needs to be matched for semantic orientation. The evident outcomes also help in time reduction aspect where huge volume of data need to be processed swiftly. This noteworthy contribution brought up significant knowledge and direction for domain experts. In the future, the proposed technique will be explored for other types of textual data across different domains.

## 1 Introduction

Over the last decade, sentiment analysis has become an emerging field of interest in the research community [1]. People express their opinions about products, celebrities, companies, movies, etc. using social media. The process of mining sentiments, opinions, attitudes, or feelings from text is called sentiment analysis. This involves identifying and analyzing positive and negative attitudes in the user-written text. The applications of sentiment analysis include predicting the election results based on user tweets or a quality improvement of a product using customer tweets [2]. Various terms for sentiment analysis are used in the research community, such as "opinion mining,” "sentiment classification,” "opinion analysis,” "opinion classification,” "sentiment mining,” "opinion extraction" and "subjectivity analysis" [3, 4]. Smith [5] defined sentiment analysis as; "Sentiment Analysis is the computational evaluation of documents to determine the fine-grained emotions that are expressed."

A large amount of data is generated by social media platforms, including Twitter, Facebook, LinkedIn, Instagram, Pinterest, and Flicker. This data plays a vital role in decision-making for different organizations such as for businesses, sports clubs, and political, social, and marketing organizations. Such a huge amount of data can figure out a very important question i.e., "what people think" and this question is very important to take a step forward or make a decision [6].

Founded in 2006, Twitter is one of the most popular microblogging platforms where users can post messages called "Tweets". More than 330 million active users are currently reported [7]. The Twitter platform is fundamentally based on a short message service, in which a user can send a message comprising 280 characters. Hashtags that appear popular on Twitter have become a trend. This trend could be a topic that most Twitter users discuss and share. These trends can be on country or international level among worldwide users. Twitter is used by almost all the popular figures worldwide. Even country representatives use Twitter to communicate information with each other. One such incident happened recently, when Iran and the American government used Twitter to openly threaten each other [8]. These two countries had no formal diplomatic relations, and thus they used Twitter to convey their messages in front of the entire world. The power of Twitter can also be seen in the fact that #metoo has changed the entire environment of the Hollywood film industry.

There are several other examples that show the power of microblogging platforms, including disaster recovery and breaking news sources. Twitter has created a distinctive idiom owing to the restriction of text size and sharing mechanisms [9]. Unlike the traditional writing styles of blogs, news, and other social media posts, Twitter messages are much shorter in size yet have a powerful impact on society. The language of tweets seeks a lot of attention in the research community, as the restriction of short text allows grammatical changes, containing more abbreviations, misspelled words, and emoticons. These forms of text are usually avoided in traditional text writing such as newspapers, emails, and blogs [9]. The underlying importance of Twitter text is significant for various applications including sentiment analysis, text classification, communication studies, machine translation, and sociology.

Researchers have invested considerable effort in bringing valuable knowledge to the research community. The main aim of researchers is to improve the accurate mining of public sentiment. Four different types of approaches were considered for sentiment analysis. These are heuristic-based, rule-based, lexicon-based, and machine learning-based approaches. In rapidly evolving machine-learning-based approaches, parts of speech (POS) play a vital role in feature selection and classification tasks. Several studies have been conducted to analyze sentiments on Twitter [10–14] based on machine learning classification.

Machine learning classification algorithms provide an automatic method for categorizing textual messages. This process is known as text-classification. The text in the documents is represented in the form of feature vectors. Various textual, temporal, and geographical features are associated with text documents. Several methods can be used to identify, select, and extract the classification features. For sentiment classification, various classifiers are used by the research community, such as support vector machine [15–20], naive Bayes [21–24], maximum entropy [13, 25], neural network [26], AdaBoost [27], and random forest [28]. Numerous classifiers have reported varying accuracies using various classification features. This study investigated the genuine possibility of various features performing better on comparable terrains using classifiers reported by the research community.

Different metrics are considered for evaluating classifier performance, such as precision, recall, F-measure, and overall accuracy. Sentiment analysis has become a challenging task because the language used in tweet text often contains many impurities with the actual sentiment. This is addressed by applying the part-of-speech (POS) tagging approach [23, 25, 27, 29]. This study focuses on the extraction of sentiment using various part-of-speech (POS) entities such as verbs, adverbs, nouns, pronouns and adjectives. Exploring these language entities is important for understanding intended opinions in text form. Numerous state-of-the-art POS taggers have been proposed and utilized by different researchers [30]. The research community frequently utilizes the Stanford POS tagger [31], whereas POS tagger-like CLAWS are becoming a focus in the domain of sentiment analysis [32]. CLAWS offers a more extensive list of English language tags with 96%–97% accuracy [33]. A descriptive list of Adjectives and Adverbs Tags from both the UCREL [33] and Stanford’s natural language processing (NLP) research groups [31] is provided in following section.

The rest of the paper is organized as follows: Section 2 presents a literature review, and Section 3 elaborates the research methodology. Section 4 discusses the experiments and results. Finally, Section 5 concludes the research effort.

## 2 Literature review

Sentiment analysis is a popular research area for analyzing different trends and discovering useful insights based on microblog data. There has been considerable research on Twitter, the biggest microblogging platform. Let us review some research efforts in detail.

Wakade et al. [10] addressed the problem statement stated as follows: Given a collection of tweets related to a specific subject, how do we come up with a classifier for labeling the sentiment of new tweets as positive, negative, or neutral?". Tweets concerning Microsoft and I-Phone were gathered from a publicly available dataset. These data were preprocessed by eliminating "stop words, " prepositions, and pronouns. Stemming was performed to reduce and clean the datasets. For classification purposes, the dataset was passed to J48 and naive Bayes algorithms, where the J48 algorithm outperformed naive Bayes.

Agarwal and Mittal [11] proposed a technique to identify terms (normally shorter terms) in Twitter messages to determine the contextual polarity for classification. Linguistic-based feature sets, such as nouns, verbs, adverbs, and adjectives, are generated by combining four specific lexicons and other features. Then, a Support Vector Machine (SVM) classifier was applied to these feature sets to classify the identified contextual polarity in the messages.

Sahayak et al. [12] used various machine learning classifiers to propose a technique for classifying tweets. These classifiers include support vector machines, naive Bayes, and the maximum entropy. For classification features, researchers extracted n-grams, such as unigrams, bigrams, n-gram combinations, and POS features. It was observed that the machine learning technique performed well for sentiment analysis tasks.

Saif et al. [13] improved the task of tweet classification by using a semantic concept-based technique. In this technique, the semantic concepts of entities are combined into a training set as additional features. Researchers have used three distinct methods for incorporating these additional features into the training set of naive Bayes. The use of POS and unigrams as baseline standards is also depicted. The experiments concluded that semantic features outperformed the POS and unigrams baseline models in identifying sentiment polarities.

Amolik et al. [14] experimented with movie reviews related to tweets and performed sentiment analysis. The feature vector is created after the preprocessing phase based on specific Twitter features. Later, classification is performed into positive and negative classes by applying SVM and naive Bayes classifiers. The results showed that SVM performed with 75% accuracy, and naive Bayes produced 65% accuracy.

Apart from Twitter-based research, in a recent research survey, it was stated that natural language processing (NLP) began in the 1950s, but very little attention had been paid until 2005 [1]. Survey researchers have claimed that sentiment analysis has evolved and can be considered a specialized branch of machine learning that also deals with psychology and sociology. Let us critically review and discuss the evolving domain of sentiment analysis from 2005 to 2020 and evaluate the criteria primarily followed by domain experts for sentiment analysis tasks.

Wilson et al. [34] proposed a method for determining the contextual polarity using phrase-based sentiment analysis. The phrases were classified into neutral and polar, followed by the second step, in which all polar phrases were further distinguished based on their contextual polarity. For contextual sentiment analysis, this study used an annotated dataset in which the annotator classified the phrases into positive, negative, and neutral classes based on contextual sentiment rather than on subjective sentiment. For neutral-polar classification, 75.9% accuracy was achieved, whereas for polarity classification, this study achieved an overall accuracy of 67.5%.

Whitelaw et al. [15] identified and extracted appraisal groups where each group contained a set of attribute values in several task-independent semantic taxonomies. These attributes were then combined with traditional bag-of-words (BOW) features using a support vector machine (SVM) classifier to achieve an overall accuracy of 90.2%. Four appraisal groups were proposed: attitude, orientation, graduation, and polarity. These four types of attributes were extracted from the movie review dataset and used for sentiment classification purposes.

Vincent et al. [16] proposed a method to determine whether a text is a review and whether the review is positive or negative. The SVM classification method was used for the classification tasks. For the prior task, 99% accuracy was achieved via 10-fold cross-validation, whereas 90.5% and 86.1% accuracies were achieved for the later task using the two datasets. We employed two datasets for sentiment classification. By adding adjective polarity information and discarding objective materials, the SVM classifier attained 86.2% and 90.5% overall accuracy on Datasets B and A, respectively.

Chesley et al [17], in a similar study used adjective and adverbs features for sentiment classification on blog data. This study focused on: a) the role of classes of verbs in identifying blogs’ sentiment and b) categorization of adjectives expressing polarity using online lexical resources. The feature set used for classification comprises various types, including objectives, subjective, textual, adjectives, positive verbs, and negative verbs. Using the SVM classification method and annotated dataset, all the combined features achieved accuracies of 90.9%, 89.93%, and 91.2% for the objectives and positive and negative classes, respectively.

In the approach of Godbole et al. [35], a sentiment score is assigned to each entity residing in the text. The proposed system operates in three steps: In the first step, an algorithm is proposed to extract words with sentiments. In the second step, sentiment aggregation is performed, followed by the third step, in which the polarity score is assigned to each term in the lexicon and subjectivity represents the proportion of sentiment to the frequency of occurrence. The overall score of an entity at a certain threshold determines whether it is cited as positive or negative.

Benamara et al. [36] proposed adjective- and adverbs-based sentiment analysis. They proposed three axiomatic scoring methods, based on adjectives and adverbs. Each sentence is scored from -1 to 1, where -1 represents an extreme negative sentiment and 1 represents an extreme positive sentiment. The degree of adverb based on the power of making an adjective negative or positive was grouped into five categories. These include “adverb of affirmation,” “adverbs of doubt,” “strong intensifying adverbs,” “weak intensifying adverbs” and” negation and minimizers”. The algorithm proposed in this study attained a high Pearson correlation coefficient (0.47) compared to human subjects (0.13, 0.19).

Denecke [37] demonstrated a sentiment-analysis task within a multilingual framework. The SentiWordNet lexical recourse was used for this purpose. Standard translation software was used to translate non-English text into English. The aforementioned lexical resource was used to assign polarity scores to the adjectives found in the text of the document. The final score of the documents was determined by the occurrence of polarity terms in the text. The author argues that existing translation technologies and lexicon resources can be used for multilingual sentiment analysis. While this approach is straightforward, the author did not consider the problems associated with translation; however, word-by-word translation could change the meaning of a sentence or context.

Annett and Kondark [38] investigated both lexical- and machine-learning-based approaches for sentiment classification of movie reviews. In the prior experimental setup, the blog post was treated with standard text pre-processing techniques, and the extracted words were then searched in two dictionaries: General Inquiries and Yahoo words. A polarity score was assigned to each term appearing in the document text. The overall polarity of a document depends on the availability of positive or negative terms in the text. In the machine learning-based approach, the feature set included a) “number of positive terms”, b) “the number of negative terms”, c) “number of negating words” and d) and term frequency of popular words. The results of the lexical experiment setup resulted in a high accuracy of 60.4%, whereas the SVM for the machine learning-based experiment attained 77.1%.

Boiy and Moens [25] tackled sentiment analysis as a classification task and applied machine-learning-based sentiment classification to English, Dutch, and German texts. Support vector machine (SVM), multinomial naive Bayes (MNB), and maximum entropy (ME) classifiers were employed for classification purposes. Precision, recall, f-measure, and accuracy measures were used to evaluate the performance of the classifiers. The SVM and ME attained high accuracies of 86.35% and 87.40%, respectively, using unigram and subjectivity features.

Narayanan et al. [39] investigated conditional sentences for Sentiment analysis. Conditional sentences are defined as "sentences that describe implications or hypothetical situations and their consequences". The conditional sentences comprise two parts: a) the condition part and b) the consequent part. The author argued that the presence of polarity words in the consequent part of a sentence has a major impact on the overall sentiment of a sentence, with a high accuracy of 67.3%.

Pak and Paroubek [18] performed sentiment analysis on the collected dataset, and the results revealed that objective text contained more proper and common nouns than subjective text. Subjective text contained simple past tense with the base form of the verb, whereas objective text tended toward the third person. They further added that for the representation of opinion or sentiment, the user mostly used superlative adjectives, whereas for fact-telling, the user used comparative adjectives. The SVM classifier is used for classification purposes, where bigram features perform well in sentiment detection. A similar sentiment lexicon, recourse SentiWS in German, was developed by Remus et al. [40].

Agarwal et al. [41] proposed various methods for classifying tweet data into polarity classes. Three types of models were used for sentiment classification: unigram, feature-based, and kernel-based models. The kernel-based model represented the tweets in a tree-like structure, whereas the unigram model used a base model. The feature-based model significantly reduced the feature space and execution time. The kernel-based model outperformed both the methods.

Kouloumpis et al. [27] investigated an Ada-Boosting technique for sentiment classification. HASH, EMOT, and ISIEVE datasets were used in this study. After standard pre-processing techniques are applied to the datasets, several types of features are extracted, including n-gram, lexicon, part-of-speech, and microblogging features. The combination of n-grams, lexicons, and microblogging features achieved a higher accuracy.

Wang et al. [21] developed a real-time Twitter sentiment analysis system to analyze tweet sentiment related to the presidential election in 2012. The Amazon Mechanical Turk (AMT) crowd-sourcing platform was used to annotate the training set. The naive Bayes classification method is used to train the annotated dataset for classification purposes, which has provided promising results.

Balahur et al. [42] investigated the implicit expression of emotions using sentiment analysis. Tweets or product reviews present sentiment clues in the form of polarity terms. However, sometimes user text contains implicit sentiments. In such cases, the user does not present their opinions directly or using explicit polarity terms. Implicit emotions include fear, anger, disgust, sadness, guilt, joy, and shame. In this study, we used the ISEAR dataset from Psychology. The EmotiNet knowledge base of emotion is used to extract emotions from the text and combine it with other techniques to prove appropriate results.

Htay and Lynn [29] used nouns, adjectives, adverbs, and verbs to extract opinions regarding a specific feature of any product from user reviews. They argue that the extraction of product features and opinions is an adequate source to generate a meaningful summary of product reviews. For feature extraction, this study used a POS tagger to extract nouns and phrases. The nearby adjectives and adverbs are associated with product features representing the users’ opinions. The proposed approach attained recall, precision, and F-scores of 0.85, 0.73, and 0.79, respectively.

Gamallo et al. [22] proposed a sentiment analysis system based on a naïve Bayes classification algorithm to detect sentiments in Spanish tweets. In their study, they experimented with binary and multi-classification and showed that good results could be achieved for sharp polarity categories, that is, positive and negative. In multi-class classification, several classes of neutrals have been suggested, such as strong, weak, and average neutral class. The part-of-speech pattern was also tested. These parts of speech included adjectives, nouns, adverbs, verbs, and pronouns, which showed 67% overall accuracy for multi-class classification.

Santos and Gatti [26] considered deep neural networks for sentiment-classification tasks. Bag-of-words (BOG) approaches combined with prior knowledge may be an adequate solution. This study proposed Convolutional Neural Network (CNN), exploiting "character to sentence level information" for short text sentiment analysis. The proposed CNN method is evaluated on two well-known sentiment analysis benchmarks: a) "Stanford Sentiment Treebank (SSTb)" and "Stanford Twitter Sentiment Corpus (STS)". A binary classification (negative/positive) accuracy of 85.7% was achieved for the SSTb corpus, whereas, for the same corpus, 48.3% accuracy was attained in terms of fine-grained classification. The proposed deep CNN predictor achieved 86.4% accuracy for the STS dataset.

Kiritchenko et al. [43] used short text documents for sentiment evaluation. The proposed sentiment analysis system was ranked at the top in the SamEval-2013 task. The proposed system detects sentiment at the message and term/phrase levels. The evaluation was performed using two datasets: a) tweet dataset and b) short messages (SMS). They suggested a tweet-specific lexicon as the best feature to be used in supervised and unsupervised environments. Furthermore, two lexicons were built: a) negated context terms and b) affirmative context terms. The features extracted from the aforementioned lexicons exhibited high performance. They also showed that the proposed system could process 100 tweets per second in a big-data environment.

Fang and Zhan [23] performed sentiment analysis on Amazon product reviews. The proposed method is comprised of several tasks including "sentiment sentence extraction,” "POS tagging,” "sentiment phrases identification,” "sentiment scoring,” "feature vector generation" and "sentiment categorization". Sentences are extracted from a dataset comprised of 5.1 million Amazon product reviews where sentiment terms appear. A sentiment score is assigned to each term based on its occurrence in different star-level sentences. Vector features were generated for each review and three classification algorithms were used: random forest, naive Bayes, and SVM. The results revealed that random forest attained a high f-1 score for sentence-level categorization, whereas SVM attained a high f-1 score for review-level categorization.

Agarwal et al. [44] proposed sentiment analysis based on ConceptNet ontology and context information. The concepts extracted using ontology-based methods were further treated with polarity extraction using a contextual polarity lexicon. Mertiya and Singh [24] used naïve Bayes for review classification as truly polarized and falsely polarized. This is followed by another step, in which falsely polarized records are treated with an adjective analysis. These were further classified into positive and negative tweets. In the adjective-based analysis, if the tweet contained two negative adverbs/adjectives or two positive adjectives/adverbs, the final polarity was considered as negative or positive, respectively.

Bouazizi and Ohtsuki [28] performed sentiment classification by using seven sentiment classes. The authors proposed a tool called SENTA for sentiment classification, with the provision of a user interface. Users can select the number of features for an adequate search. The SENTA tool utilizes the power of the KNN algorithm for classification. The overall accuracy achieved by SENTA was 60.2% for multi-class categorization, whereas a high accuracy of 81.3% was achieved for binary classification. For ternary classification (i.e., positive, negative, and neutral classes), the SENTA tool achieved 70.1% accuracy.

Manek et al. [20] emphasized the importance of the Gini index for feature selection in sentiment classification of movie reviews. The proposed method comprises several steps, including data acquisition from various movie websites, pre-processing the dataset with standard pre-processing methods, Gini index for feature selection, use of top k attributes for classification, and finally, the SVM classification method to classify the reviews into positive and negative categories. The proposed method achieved an accuracy of 96.95% on the acquired movie review dataset.

Hu et al. [45] adopted a text-summarization technique for opinion mining in hotel reviews. The proposed method worked in five steps: a) reviewed acquisition from TripAdvisor, b) pre-processed the reviews using pos tagging, stop word removal, pos filtering, and sentence selection; c) in the third step, the importance of a sentence is calculated using author credibility, review time, review usefulness, and review sentence; d) in the fourth step, sentence similarity is calculated using sentiment similarity and content similarity; e) in the final step, unsupervised machine learning technique K-medoids clustering is applied to partition the sentences. The proposed approach achieves promising results.

Zheng et al. [19] investigated a feature selection method for sentiment classification of Chinese text. They suggested that 4-POS-grams achieved high performance on Chinese texts. Various methods have been introduced for feature selection in the text corpora. These included character, BOG, and POS n-grams. In this study, n-char-gram and n-POS-gram features were selected, and document frequency (DF) was modified to select the subset of features. To assign a weight to the feature subset, researchers have used the Boolean weighting model. Online mobile-related Chinese reviews were used as the corpus, and the significance of the experiment was evaluated using the chi-squared test. The overall accuracy of 4-POS-gram using the SVM classifier was reported to be 93.6%.

Ragini [46] suggested a sentiment analysis technique to analyze disaster response and recovery by sharing people’s thoughts in pre- and post-disaster situations. For example, if an earthquake hits, people update their status within a couple of minutes, even before news breaks into the mainstream media. This study proposed a big data-driven approach in which disaster data are collected from different social media platforms. Machine learning algorithms were used to extract sentiments. For the supervised classification method, the lexicon-based approach yielded the best results.

Sánchez et al. [47] analyzed the scope of virtual reality (VR) technology using YouTube data. User sentiments are judged for contribution of VR technology for sustainability of natural environments. YouTube reviews and comments are utilized to understand which attributes of virtual reality are the most effective. Users generated data remained important for decision making about the future policy of VR companies. Positive emotions prevailed on emotions of anger or frustration with clear appreciation of VR features like video quality, VR glasses etc.

Kastrati et al. [48] learnt the public engagement on rising energy prices using sentiment analysis. Author experimented the dataset of tweets collected between January 2021 to June 2022. This approach took advantage of both a transformer-based sentiment analysis method and topic modeling in order to explore the energy trends. BERT is applied to divide tweets into neutral, positive, and negative, and then a topic modelling based on BERTopic and LDA is used to identify relevant sub-topics associated to energy. The findings reveal that the public sentiment towards these topics has changed the over time, particularly in 2022 when negative sentiment was dominant.

Braig et al. [49] studied the human behaviours during the pandemic of COVID-19. Authers mapped the people thinking, feeling, and acting on a sentiment dashboard. The data was acquired from SpringerLink, IEEE Xplore DL, AIS Electronic Library, ScienceDirect and ACM DL. COVID-19 related topics were investigated from 40 research articles retrieved from above sources. In order to facilitate government for decision making, mainly three classification approaches have been used lexicon-based, machine learning-based and deep learning-based methods. Using sentiment analysis, different insights from social and behavioral science helped out public health experts with guidance against the spread of COVID-19.

However, in the last couple of years, many researchers have focused solely on specialized adverbs and have produced improved results with specific types [32, 50, 51]. These researchers have focused on three to ten different types of adverbs recognized by linguistic research groups. With significant results, Chauhan et al. [32] experimented with Amazon reviews using the SentiWordNet-based lexicon approach. For their benchmark evaluation, Amazon’s star-based rating was considered within different ranges. However, researchers only evaluated a few types of adverbs as single features among the 16 distinct types of adverbs and four different types of adjectives published under the CLAWS linguistic research group [33] as shown in Table 1. Boukabous and Azizi applied a hybrid approach to improve the results of sentiment analysis on a crime-based tweet dataset. They produced better results by focusing on adjectives, adverbs, and noun-based features for sentiment analysis tasks [52]. Chen et al. also studied sentiments in hotel reviews. Their technique converted textual reviews into sentiment scores, which distinguished the actual hotel attributes that contributed to star ratings. According to their selection criteria, adjectives, adverbs, nouns, and verbs were exploited as candidate sentiment words [53].

**Table 1:** Adjectives and adverbs pos tag sets.

CLAWS POS Tags	STANFORD POS Tags
**JJ**: general adjective (blue) **JJR**: general comparative adjective (older) **JJT:** general superlative adjective (strongest) **JK:** catenative adjective (be willing to)	**JJ:** adjective (yellow) **JJR:** comparative adjective (bigger) **JJS:** superlative adjective (wildest)
**RA:** adverb, after nominal head (else, galore) **RGR:** comparative degree adverb (more, less) **RGT:** superlative degree adverb (most, least) **REX:** appositional constructions (namely) **RG**: degree adverb (very, so, too) **RGQ:** wh- degree adverb (how) **RGQV:** wh-ever degree adverb (however) **RL**: locative adverb (alongside, forward) **RP:** prep. adverb, particle (about, in) **RPK**: prep. adv., catenative (be about to) **RR:** general adverb **RRQ**: wh- general adverb (where, when) **RRQV:** wh-ever general adverb (whenever) **RRR:** comparative general adverb (better) **RRT:** superlative general adverb (longest) **RT:** quasi-nominal adverb of time (tomorrow)	**RB:** adverb (quickly, never) **RBR:** comparative adverb (faster) **RBS:** superlative adverb (fastest)

After comprehensively and critically reviewing the sentiment analysis task during the evolving period of 2005 to 2022, following Table 2 summarized the POS-based feature criteria widely followed by the research community.

**Table 2:** Critical literature review summary (✓: Yes, X: No).

Year	Authors	Feature types							
		Adjective	Adverb	Verb	Noun	Types of adjectives	Types of adverbs	Combo Of types	Negation
2005	Wilson et al.	✓	✓	✓	✓	X	X	X	✓
	Whitelaw et al.	✓	✓	✓	✓	✓	X	X	✓
2006	Vincent et al.	✓	X	✓	✓	X	X	X	X
	Chesley et al.	✓	X	✓	X	X	X	X	X
2007	Godbole et al.	✓	X	X	X	X	X	X	✓
	Benamara et al.	✓	✓	X	X	X	✓	✓	✓
2008	Denecke	✓	X	✓	✓	X	X	X	✓
	Annett & Kondrak	✓	✓	X	X	X	X	X	✓
2009	Boiy et al.	✓	X	✓	X	X	X	X	✓
	Narayanan et al.	X	X	✓	X	X	X	X	✓
2010	Pak & Paroubek	✓	✓	✓	✓	✓	✓	X	✓
	Remus et al.	✓	✓	✓	✓	X	X	X	X
2011	Agarwal et al.	✓	✓	✓	✓	X	X	X	✓
	Kouloumpis et al.	✓	✓	✓	✓	X	X	X	✓
2012	Wang et al.	✓	✓	✓	✓	X	X	X	✓
	Balahur et al.	✓	✓	✓	✓	X	X	X	✓
2013	Htay & Lynn	✓	✓	✓	✓	X	X	X	✓
	Garcia & Lanza	✓	✓	✓	✓	X	X	✓	✓
2014	Santos & Gatti	✓	✓	✓	✓	X	X	X	✓
	Kiritchenko et al.	✓	✓	✓	✓	X	X	X	✓
2015	Fang & Zhan	✓	✓	✓	X	X	X	X	✓
	Agarwal et al.	✓	✓	✓	✓	X	X	X	✓
2016	Bouazizi & Ohtsuki	✓	✓	✓	✓	✓	✓	X	✓
	Mohit & Ashima	✓	✓	X	X	X	X	X	✓
2017	Manek et al.	✓	✓	✓	✓	X	X	X	✓
	Hu et al.	✓	✓	X	✓	X	X	X	X
2018	Zheng et al.	✓	✓	✓	✓	X	X	X	✓
	Ragini et al.	✓	✓	X	X	✓	✓	X	✓
2019	Haider et al.	X	✓	X	X	X	✓	✓	✓
2020	Chauhan et al.	X	✓	X	X	X	✓	X	✓
2021	Boukabous & Azizi	✓	✓	X	✓	X	X	X	✓
2022	Chen et al.	✓	✓	✓	✓	X	X	X	✓

As is evident from the literature, for the last few years, Adjectives and Adverbs have mainly focused on textual data for grabbing actual sentiments. However, it was discovered that none of the studies conceptualized all important existing Adjectives and Adverbs-based features on a single dataset. Thus, a vital approach is required which can comprehensively analyzes the types of Adjectives and Adverbs reported in the diverse literature such as in CLAWS-C7. Therefore, following research questions (RQs) are formulated for further exploration of identified research gap.

**RQ#1**: How do different types of Adjectives could be used to get good accuracy for Twitter sentiment analysis?

**RQ#2**: How do multiple types of Adverbs contribute to the accuracy of sentiment classification on Tweets?

**RQ#3**: How do significant Adjective and Adverb Types work together in combination for accurate sentiment mining?

After conducting experimentation, all the research questions are answered with detailed analysis. Investigating these research gaps can reduce multi-directional efforts and bring valuable knowledge and direction to the scientific community.

## 3 Methodology

**Fig 1:**
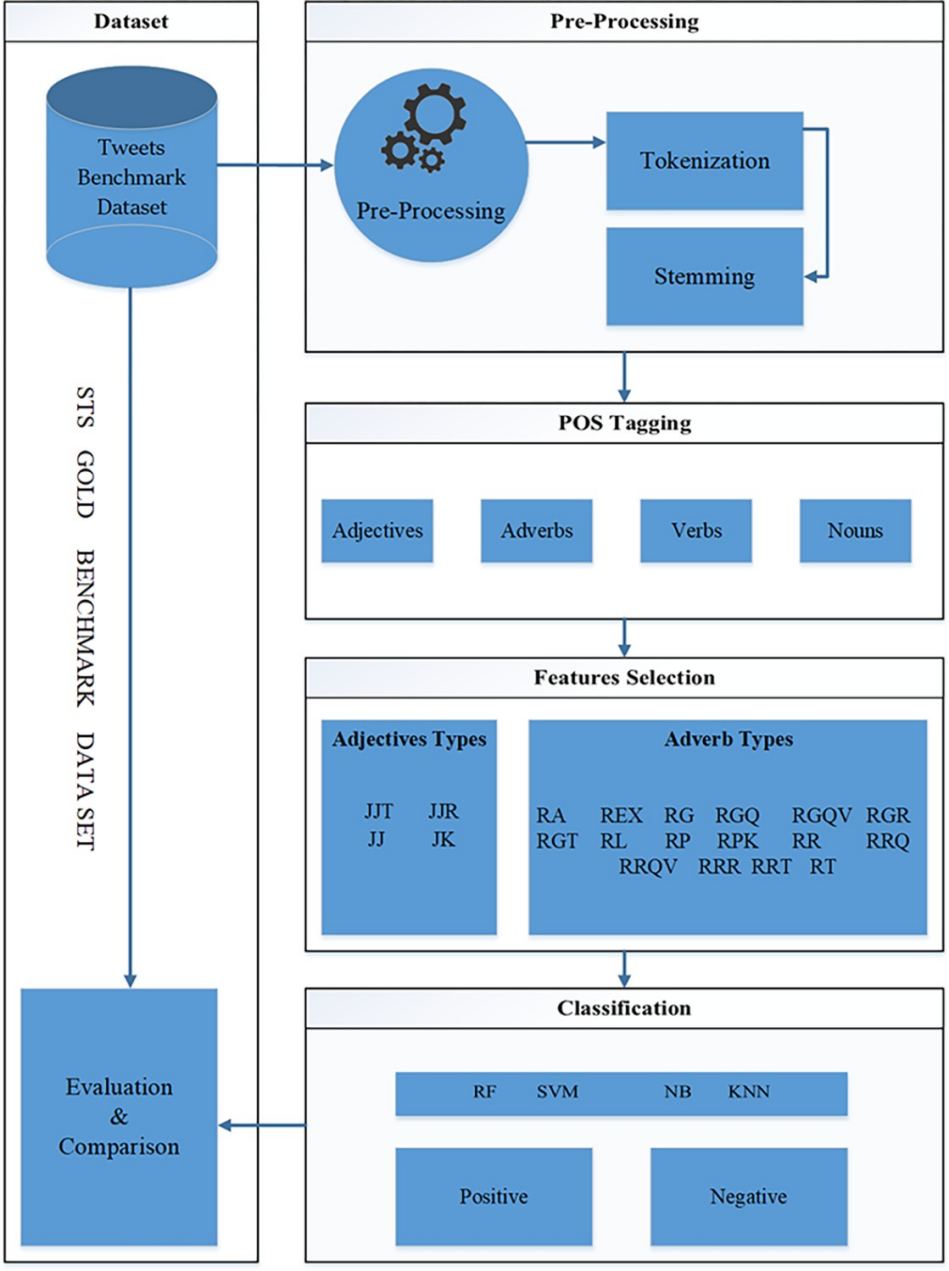
Proposed research methodology.

The proposed methodology comprises four main components: data acquisition, preprocessing, classification, and evaluation. For sentiment analysis, different forms of data are examined by the research community, including Tweets [10–14], movie reviews [15, 20, 38], Amazon product reviews [23, 32], TripAdvisor Reviews [45], Short Messages [43], and Hotel Reviews [53]. This research methodology considered a renowned dataset of tweets for detailed experimentation and evaluation. Tweets can also be acquired by a tweet crawler using the Twitter API. The Twitter API is publicly accessible for collecting tweets based on the given hashtag. The acquired tweet dataset must be preprocessed for numeric, symbolic, slang, and emojis based on short information that is commonly used by authors. This pre-processing step was vital for capturing the actual sentiments. Then, classification was applied to classify public sentiments into positive and negative classes, as annotated by expert users. This involved feature extraction and selection techniques for training the classification model. Finally, the trained classification model was evaluated using an annotated benchmark dataset. Fig 1 depicts the complete system architecture of the proposed research methodology in detail.

### 3.1 Data acquisition

Some renowned datasets are publicly available, specifically for sentiment evaluation, such as the Stanford Twitter Sentiment Corpus [27], SemEval 2015 [54], SemEval 2016 [55], and CrowdData. For the proposed research, we used a Twitter-based dataset that has the maximum availability of POS features under the CLAWS tag set. Thus, a subset of Stanford Twitter sentiments known as the STS-Gold dataset is appropriate for experimentation and the analysis of POS features [13, 56, 57]. This dataset was constructed by domain experts to address the limitations of previously known renowned datasets. This dataset contains more than 2000 tweets with an inter-rater reliability (IRR) of 1. This publicly available dataset has been cited by almost 300 studies showing the data reliability.

### 3.2 Pre-processing and POS tagging

**Fig 2:**
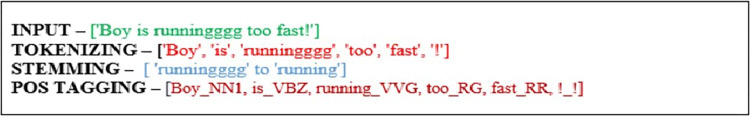
A brief example of pre-processing using NLTK API.

After acquiring the tweet dataset for experimentation purposes, pre-processing became necessary for the removal of stop words, numeric data, special characters, and emojis. The example of stop words are such as “is”, “this”, “have”, “55”, “#”,”?” and “ϑ” etc. This pre-processing phase remained necessary, as this information does not contribute to the overall sentiment of a tweet rather than textual noise. In preprocessing phase, the natural language processing (NLP) technique is applied for better mining of sentiments. The Natural Language Toolkit (NLTK) in Python language is a popular package that offers text processing libraries for tokenization, stemming, and POS tagging tweets. Tokenization refers to the splitting of text into n-grams, whereas stemming is the removal of morphological affixes from words such as "Writing" is stemmed as "Write". POS tagging is a process in which each tokenized word is tagged as part of speech (POS). POS contains different tags, such as adjectives, adverbs, verbs, and nouns. For Pre-processing different hyperparameters are adjusted; *preserve_case* is disabled as *case* of tweets does not contribute for sentiments; *strip_handles* and *stopwords* are enabled in order to exclude users’ names and stop words from tweets. Similarly, *Snowball* stemmer is utilized for stemming purpose. Fig 2 presents an example view of the preprocessing method.

### 3.3 Feature selection and classification

For classification purposes, feature extraction and selection techniques were implemented step-wise. For the feature extraction technique, part-of-speech (POS) tagging is performed by a CLAWS tagger, which is quite extensive, as shown in Table 1. The tagging of adjective- and adverb-based textual terms was manually verified, which supports the implementation of the forward feature selection (FFS) technique. FFS was applied to make classification tasks more effective and focused. As this research intend to measure the positive and negative polarities, binary classifiers are required. Finally, renowned binary classifiers are applied, which are mainly SVM, Naive Bayes, Random Forest, and K-Nearest Neighbors. SVM is a supervised machine learning algorithm for binary classification. For linear data, the SVM generates a hyperplane that bisects data in two classes with a maximum margin between them. For nonlinear, SVM can play smartly with dot products of vectors, which easily sidestep the expensive computation. Naive Bayes is another popular binary algorithm that follows probability theory. It calculates the probability of each label for a given instance and the tags with the highest probability. It is frequently applied to text classification problems for effective analysis. The k-nearest neighbor (k-NN) algorithm is a supervised statistical pattern-recognition algorithm. A data point is classified by measuring the distance to the nearest trained point, which determines the classification of samples in binary classes. The nearest neighbor is very simple but sometimes computationally intensive. Random forest (RF) is a classification algorithm based on decision trees. A decision tree is a supervised learning algorithm applied for binary classification, which orders classes at a precise tree level. Random forest utilizes subsets of decision trees, which makes them faster. Python packages were utilized for the aforementioned classifiers during the experimentation phase.

### 3.4 Evaluation and comparison

To analyze and evaluate the experimental results, a standard performance measure, that is, the accuracy score, was applied with their respective class support division as follows:

Recall is defined as:

$$Recall=\frac{True\;Positive}{True\;Positive+False\;Negative}$$

Precision is defined as:

$$Precision=\frac{True\;Positive}{True\;Positive+False\;Positive}$$

Accuracy score is defined as:

$$Accuracy=\frac{True\;Positive+True\;Negative}{True\;Positive+True\;Negative+False\;Positive+False\;Negative}$$

Based on these metrics, the evaluation was performed using recall, precision, and accuracy metrics. These evaluation metrics are widely used by the research community in sentiment analysis. The accuracy obtained by the proposed methodology demonstrates the potential of each exploited feature, which is significant for domain experts. The proposed model is also compared with state of the art Probabilistic and Lexicon based models [56, 57].

## 4 Results and discussion

This section discusses the experimental results of the proposed research methodology. To exploit the potential of adjective and adverbs-based features with their types and combinations, the classification task is categorized into three phases. Firstly, adjectives and adverbs are classified to measure their accuracies; secondly, result-oriented types of Adjectives and Adverbs are classified for better accuracies; and lastly, the combinations of Adjective and Adverbs types are examined for a comprehensive analysis. These phases are helpful to answer our research questions subsequently.

The acquired dataset contained more than 2000 annotated tweets for experimentation. Each tweet was correctly labeled into binary classes, that is, positive or negative, by [13, 56, 57]. The small number of tweets in the dataset contained different stop words, punctuation, and emotions, which were preprocessed as mentioned in the previous section. In the POS tagging stage, based on the CLAWS tag set, all extracted tags were manually inspected and observed with their existences.

**Fig 3:**
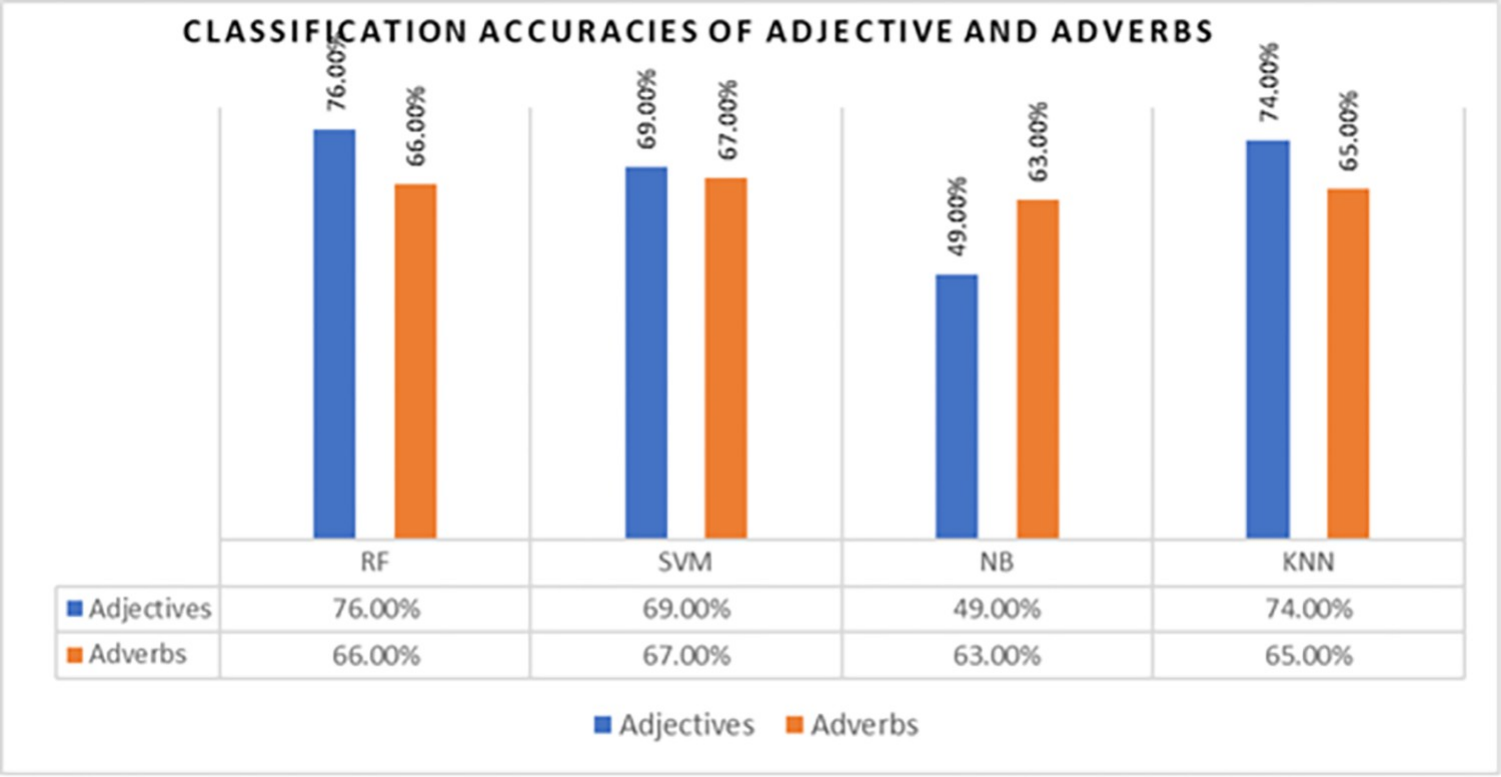
Classification results of adjectives and adverb.

In the first phase, for Adjectives and Adverbs, all classification features under 16 types of Adverbs and four types of Adverbs are collectively considered as Adverbs and Adverbs, respectively. Using these features, the classification performed using the four aforementioned classifiers: naive Bayes, SVM, random forest, and k-nearest neighbors. The obtained accuracies are shown in Fig 3.

The highest accuracies were 76% and 66% for Adjectives and Adverbs, respectively. Naïve Bayes and SVM produced low accuracies compared with random forest and KNN. Thus, Adjectives remained more effective than adverbs at classifying sentiments.

In second phase, diverse types of Adjectives and Adverb are exploited. Before performing the classification task on the acquired dataset, careful inspection was performed to identify feasible features. Around nine types of Adverbs and three types of Adjectives are found fair enough for experimentation phase. The rest of the types appeared rare; therefore, they remained insignificant for any possible contribution. It is noteworthy that the appearance of insignificant features is natural because they genuinely exist in the literature. These cannot be manually added or modified in any literature. A brief summary is provided in Table 3, where the features of the second row are neglected for experimentation.

**Table 3:** Statistics for types of adjectives and adverbs.

ADVERB TYPES	COUNTS	ADJECTIVE TYPES	COUNTS
RR, RP, RG, RT, RL, RRQ, RGQ, RPK, RRR	32,55	JJ, JJT, JJR	486
RA, RGR, RGT, RRT, REX, RRQV, RGQV	53	JK	4

In Table 4, overall accuracies of adjective and adverb types help to answer our research questions. For our first research question, Adjective types; *JJT* and *JJR* outperformed the remaining types by 83% and 78% respectively whereas in first phase, only 76% of accuracy was attained by adjectives using Random Forest. Moreover, KNN also depicted the accuracy improvement for adjective type *JJ* which is up to 77%. These results clearly indicated that Adjective types are significant for improving the sentiment classification accuracy.

**Table 4:** Classification accuracies of adjectives and adverb types with classifiers.

	TYPES OF ADJECTIVES			TYPES OF ADVERBS								
CLASSIFIERS	JJ	JJT	JJR	RRR	RPK	RGQ	RRQ	RL	RT	RG	RP	RR
**RF**	0.79	**0.83**	**0.78**	0.72	**0.65**	**0.67**	**0.77**	**0.74**	0.65	**0.64**	0.67	0.71
**SVM**	0.69	0.79	0.71	**0.74**	0.65	0.67	0.76	0.73	**0.67**	0.62	**0.68**	**0.73**
**NB**	0.55	0.62	0.42	0.34	0.65	0.67	0.58	0.36	0.65	0.45	0.37	0.35
**KNN**	**0.77**	0.60	0.66	0.67	0.63	0.65	0.64	0.64	0.49	0.64	0.57	0.62

**Fig 4:**
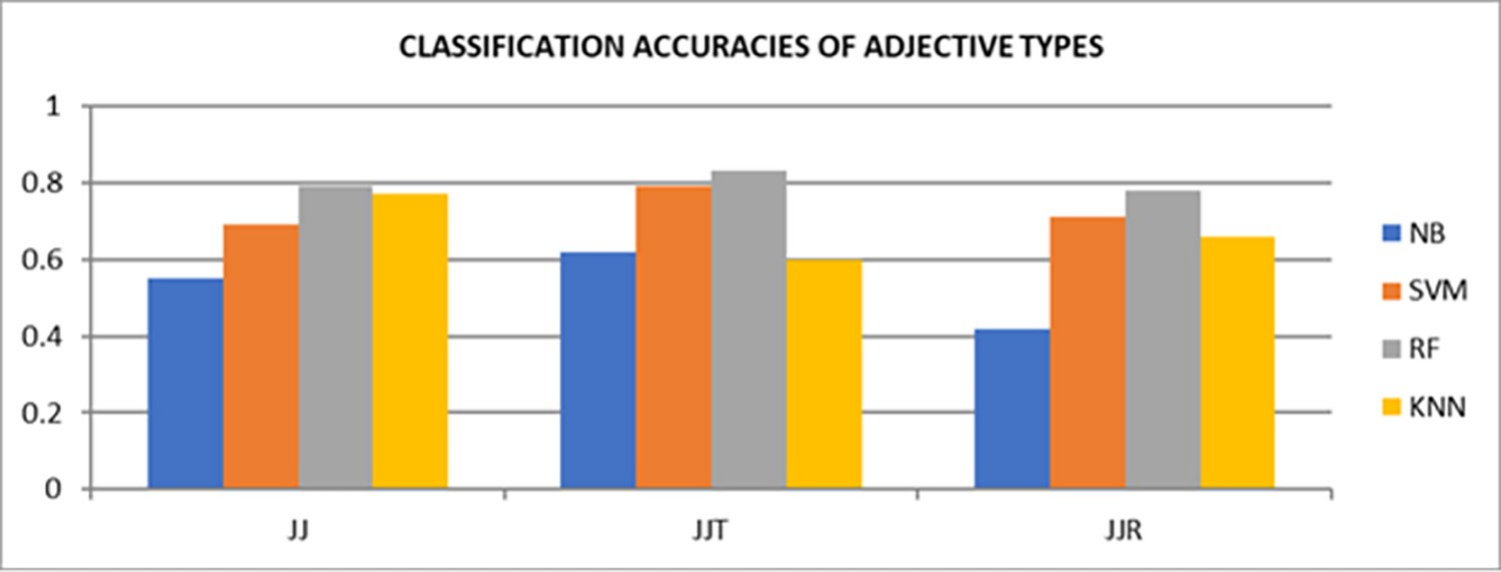
Accuracies of adjective’s types with four binary classifiers.

**Fig 5:**
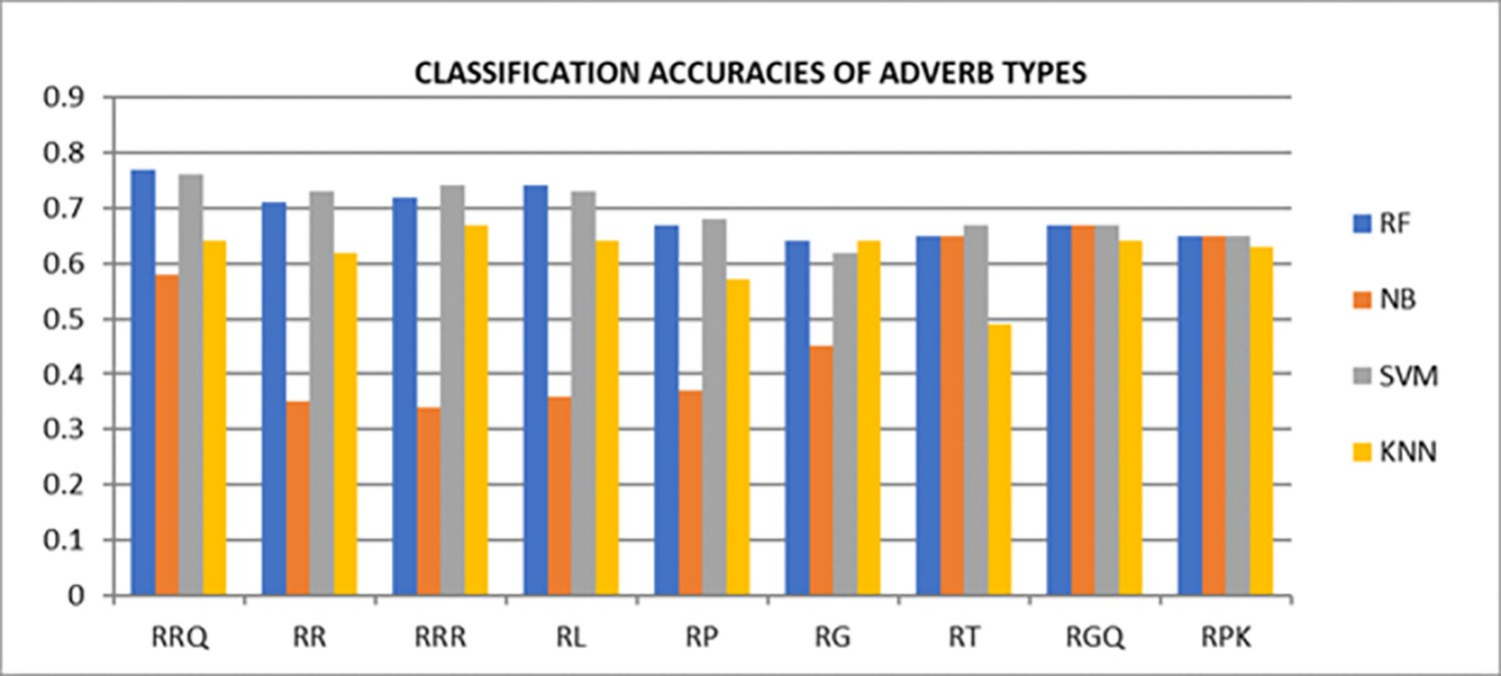
Accuracies of adverb’s types with four binary classifiers.

For our second research question, Adverbs types also showed the valuable improvement in accuracies. Adverb types: *RRQ* and *RR* outperformed the remaining adverbs types by 77% and 74% respectively using Random Forest, which was only 66% in first phase. Furthermore, SVM

also showed 73% accuracy using *RR* adverbs in contrast to initial phase which was recorded as 67%. Therefore, types of adjectives and adverbs remained vital to be exploited for sentiment classification. In contrast to Random Forest, KNN and SVM classifiers, Naïve Bayes resulted with comparatively lower accuracies. The overall comparison of each exploited types of Adjectives and Adverbs are depicted in Figs 4 and 5 respectively.

In the final phase, the forward feature selection technique (FFS) is applied to study the impact of combinations in sentiment classification. Above a certain threshold of accuracy, different types of Adjective and Adverbs were combined for an in-depth analysis. For this purpose, Adjectives and Adverb types with an accuracy more than 70% were selected from the second phase.

Therefore, on careful inspection, three types of adjectives and four types of adverbs were found to be significant and were combined for further experimentation. The results of combination obtained using all the four classifiers are listed in Table 5.

**Table 5:** Classification accuracies of adjectives and adverbs in combination.

	JJT+RR	JJT+RRR	JJT+RL	JJT+RRQ	JJR+RR	JJR+RRR	JJR+RL	JJR+RRQ	JJ+RR	JJ+RRR	JJ+RL	JJ+RRQ
**RF**	0.71	**0**.**76**	0.73	**0.76**	0.71	0.74	0.72	0.75	**0.76**	**0**.**76**	0.75	0.75
**SVM**	0.72	0.44	0.69	0.73	0.73	0.73	0.72	0.74	0.69	0.69	0.69	0.69
**NB**	0.37	0.67	0.43	0.33	0.36	0.34	0.38	0.40	0.51	0.49	0.49	0.49
**KNN**	0.66	0.71	0.72	0.73	0.68	0.64	0.66	0.63	0.68	0.70	0.67	0.72

**Fig 6:**
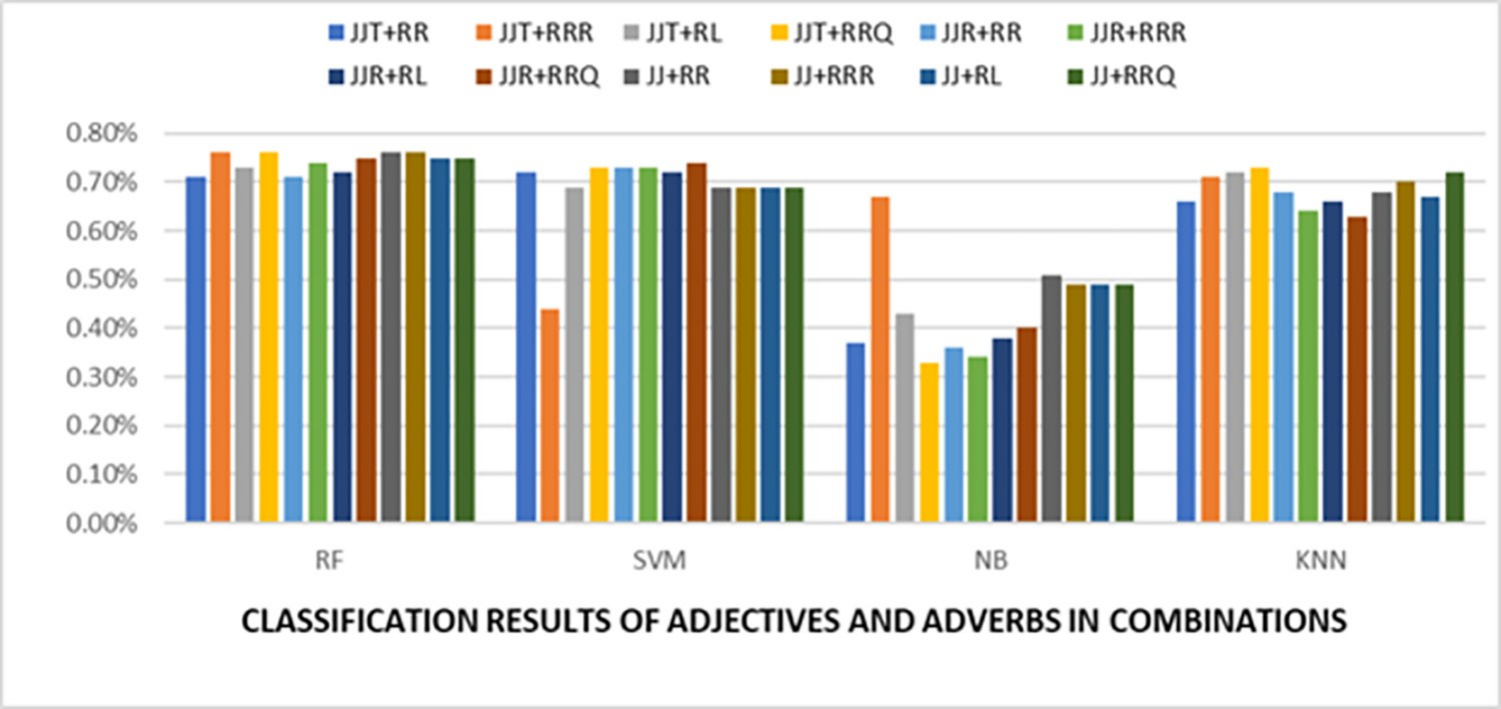
Accuracies of adjectives and adverbs in combinations.

Table 5 depicted that the highest accuracy attained by the adjective and adverb together was 76%. These combinations were *JJT+RRR*, *JJT+RRQ*, *JJ+RR*, and *JJ+RRR*. Overall random forest classifier outperformed all the other classifiers. Unlike the second phase results, K-nearest neighbors also performed well, with almost 70% accuracy for some combinations. This is noteworthy for the domain experts and can be further exploited. Following Fig 6 depicted the overall comparative results.

The proposed research approach is compared with state-of-the-art approaches. Najar et al. [56] and Thangavel et al. [57] performed Twitter sentiment analysis on STS Gold dataset. Najra et al. applied a probabilistic model named as “Smoothed Scaled Dirichlet” with ranking model principle (SSD-RM). Authors have evaluated the SSD-RM approach in terms of sparsity rates. A random generated sparse data is mixed with dataset while varying the sparsity rates. The accuracies of probabilistic model with 25%, 50%, 75% and 90% sparsity rates are shown in Table 6.

**Table 6:** Proposed model accuracies with state-of-the-art probabilistic and lexicon models.

**Probabilistic Model** (Accuracies)	**Sparsity (25%)**	**Sparsity (50%)**	**Sparsity (75%)**	**Sparsity (90%)**
	*91*.*52%*	*90*.*62%*	*77*.*14%*	*76*.*65%*
**Lexicon Model** (Accuracies)	**Text Data**	**Image Data**	**Audio Data**	**Video Data**
	*92*.*29%*	*87*.*25%*	*89*.*65%*	*85*.*85%*
**Proposed Model** (Accuracies)	**Adjectives**	**Adverb Type**	**Adjective Type**	**A&A Combination**
	*76%*	*77%*	*83%*	*76%*

Thangavel et al. exploited the multimodal contents of tweets using STS Gold dataset. Authors have analyzed the sentiments in tweets using a Lexicon based model. Different experiments are conducted using Textual, Image, Audio and Video data. The obtained accuracies on these multimodal contents are presented in Table 6.

**Fig 7:**
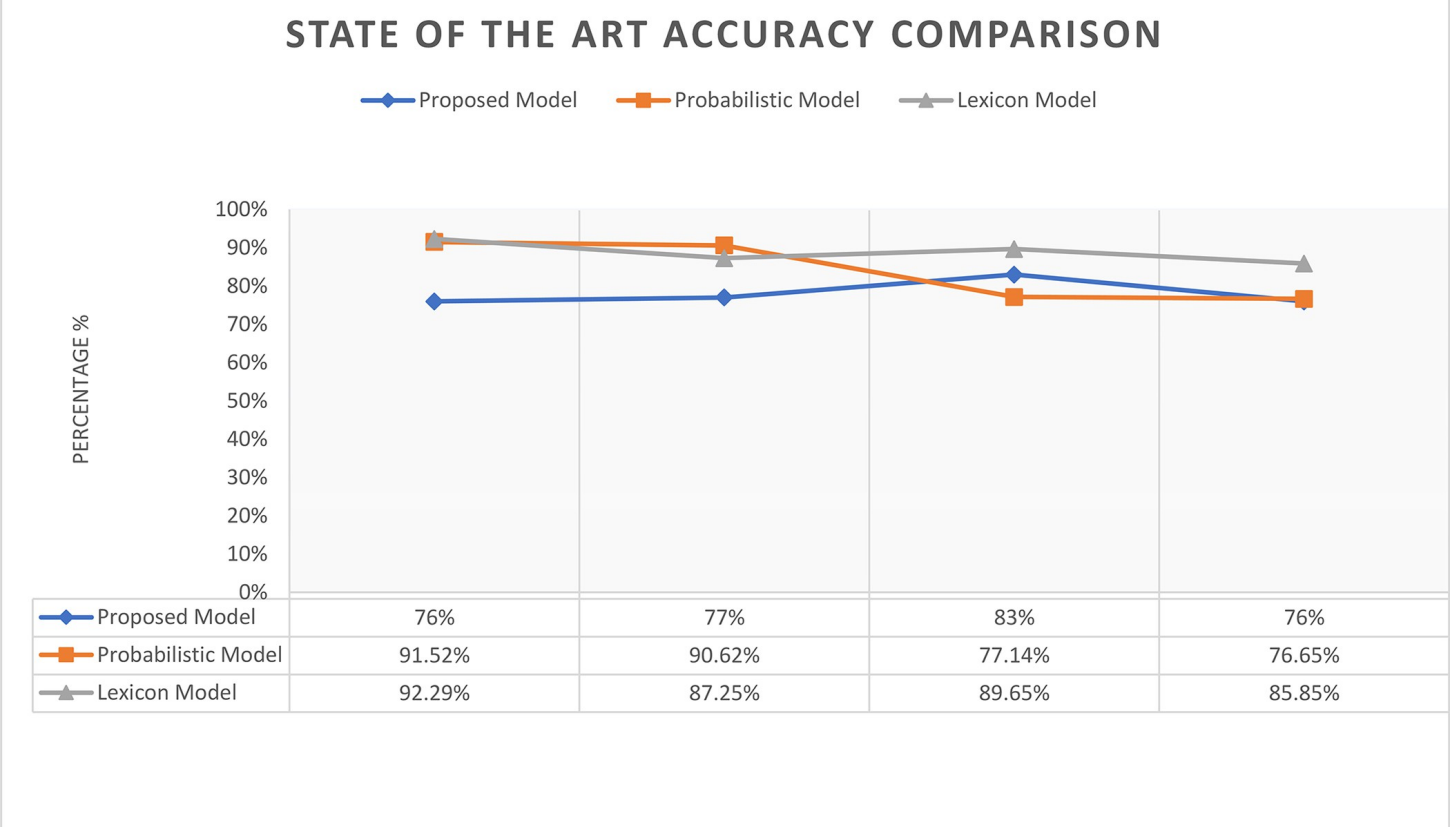
Proposed model accuracies with state-of-the-art probabilistic and lexicon models.

In order to compare the proposed model with state-of-the-art approaches on STS Gold dataset, accuracies of well performed features and classifiers are observed. The Fig 7 depicts the performance analysis of proposed model with the state-of-the-art approaches. Although simple adjective and adverb produced good accuracies using Random Forest but untested types of adjectives brought significant increase in accuracy against probabilistic-based model. With increase of sparsity rate, types of adjectives outperformed the probabilistic model. On the other hand, Lexicon based model performed well but it relies on lexicon-based dictionary. Each term needs to be matched with SentiList referred from the lexicon-dictionary table. The dependency for calculating the sentiment score is an overhead. On contrary, proposed approach revealed the underlaying potential features which already exist in any literature. Proposed model simply exploited the potential of unexplored features identified in early POS tagging phase.

## 5 Conclusion

The research community has massively focused on sentiment analysis to discover the useful insights using Twitter. In literature, researchers have noticed that adjectives significantly contribute to enhancing sentiment classification accuracy, while others argued that degree of adverbs play a crucial role in sentiment mining. According to CLAWS, Adverbs are comprised of sixteen different types whereas Adjectives are categorized in four distinct types. This research aimed to address the questions of how different types of adjectives and adverbs and their combinations impact the sentiment classification task. To achieve this goal, this study assessed the effects of diverse adjective and adverb types on humanly annotated benchmark dataset.

The research methodology followed different phases such as pre-processing, POS tagging, feature extraction, sentiment classification and evaluation. The results of the experimentation shed light on the potential of different adjective and adverb types for sentiment classification. The obtained results using adjective and adverb types improved the overall accuracy of sentiment analysis. Interestingly, JJT; general superlative adjective along with RRR; comparative general adverb outperformed the remining distinct types respectively. This combination of adjectives and adverbs exhibit a more pronounced impact among other types. In particular, this research found that JJT; general superlative adjective and JJR; general comparative adjectives achieved an impressive accuracy of 86% and 78% respectively, underscoring their significance for sentiment classification. Similarly, RRQ; wh- general adverb and RR; general adverbs played a substantial role, achieving an accuracy of 77% and 74% respectively. The proposed model showed significant results against state of the art probabilistic and lexicon-based models. Thus, it became evident that potential of specific types of Adjectives and Adverbs can improve the task of sentiment classification. This technique would benefit in time reduction aspects as in the case of Tweets, millions of tweets need to be processed quickly. In future work, the proposed technique will be applied on news articles, technology blogs, and product reviews.
